# Supplementary material for: Interactions of Fe–N–S Co-Doped Porous Carbons with Bacteria: Sorption Effect and Enzyme-Like Properties
Source: Materials (Basel). 2020 Aug 21;13(17):3707. doi: 10.3390/ma13173707 (PMC7503267; doi:10.3390/ma13173707)
Supplement: Supplementary file 1 [file materials-13-03707-s001.pdf]

materials

Article

Supplementary Materials: Interactions of Fe−N−S Co-Doped Porous Carbons with Bacteria: Sorption Effect and Enzyme-Like Properties

Andrzej Borkowski [1,*], Wojciech Kiciński [2], Mateusz Szala [2], Justyna Topolska [1] Paweł Działak [1] and Marcin D. Syczewski [3]

[1] Geophysics and Environmental Protection, Faculty of Geology, AGH University of Science and Technology, Al. Mickiewicza 30, 30-059 Cracow, Poland; topolska@agh.edu.pl (J.T.); dzialak@agh.edu.pl (P.D.)
[2] Faculty of Advanced Technologies and Chemistry, Military University of Technology, Kaliskiego 2, 00-908 Warsaw, Poland; wojciech.kicinski@wat.edu.pl (W.K.); mateusz.szala@wat.edu.pl (M.S.)
[3] Faculty of Geology, University of Warsaw, Żwirki i Wigury 93, 02-089 Warsaw, Poland; marcinsyczewski@uw.edu.pl
* Correspondence: aborkowski@agh.edu.pl

Received: 27 July 2020; Accepted: 19 August 2020; Published: 21 August 2020

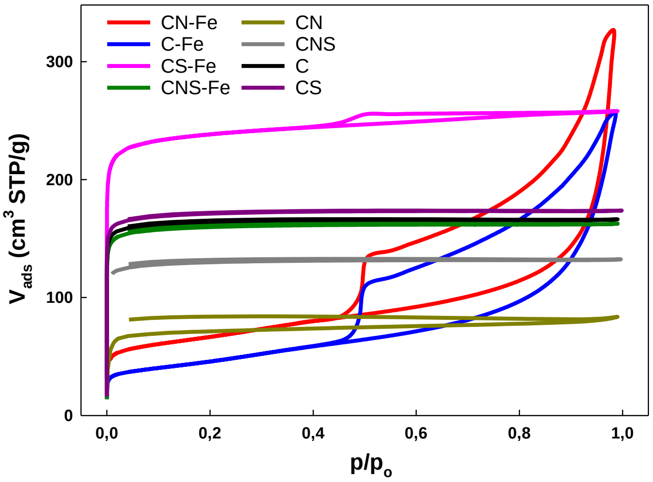

Figure S1. N_2 adsorption-desorption isotherms of the studied doped carbons.

materials
MDPI

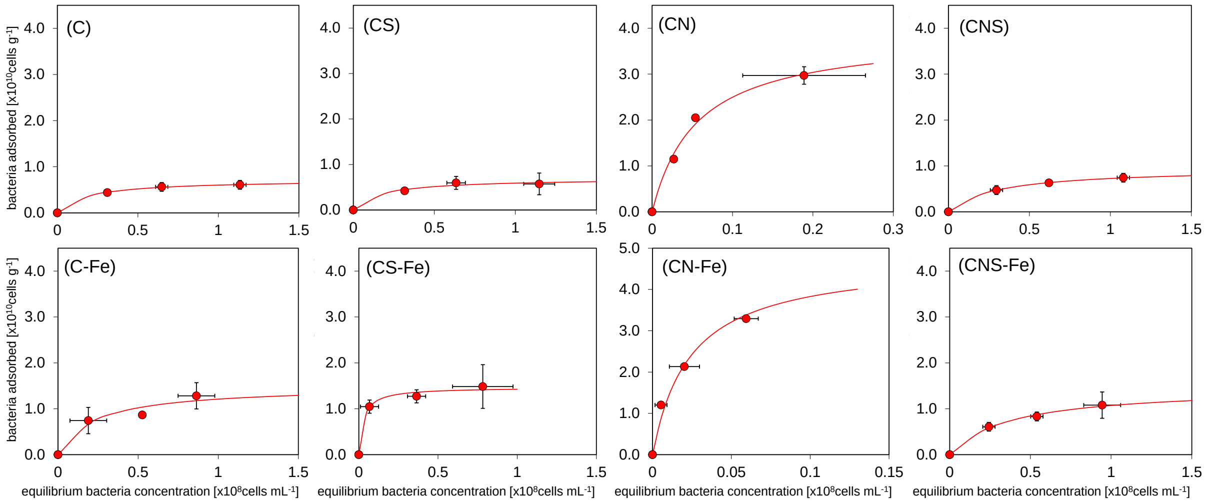

Figure S2. Adsorption isotherms of bacteria *E. coli* on the doped carbons.

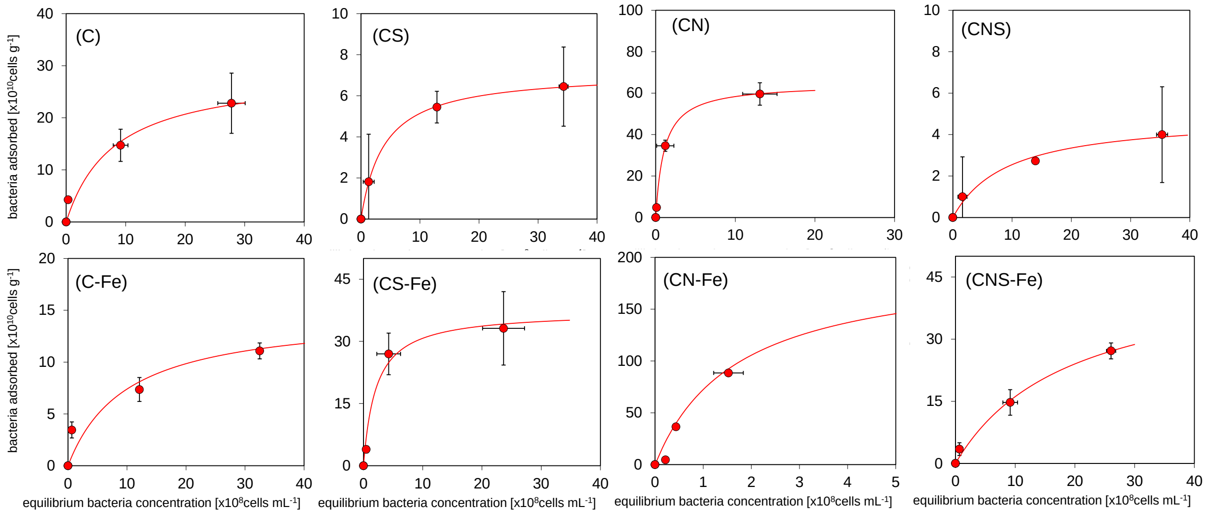

Figure S3. Adsorption isotherms of bacteria *B. cereus* on the doped carbons.

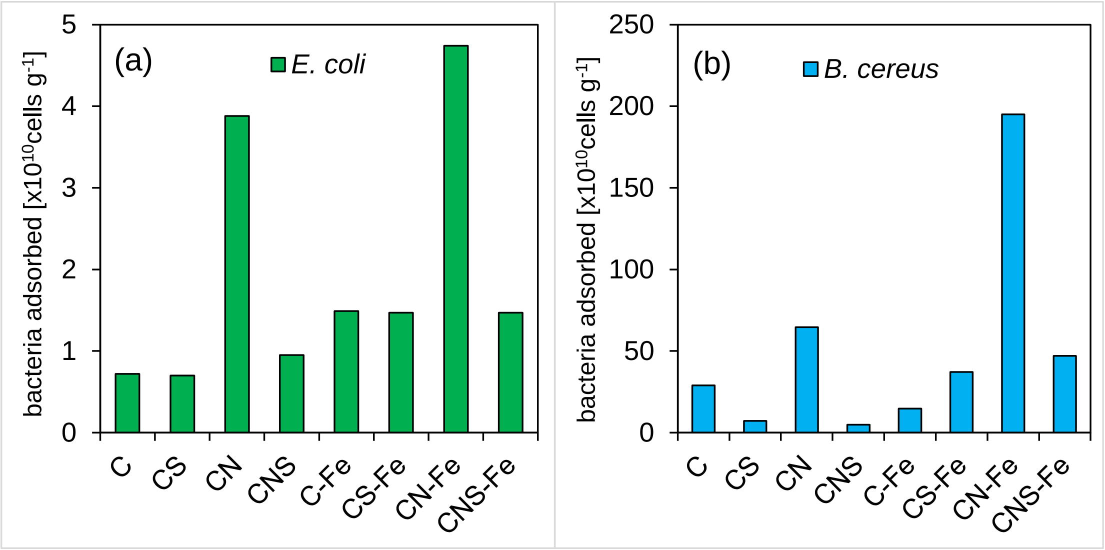

Figure S4. The maximum sorption capacity of *E. coli* (**a**) and *B. cereus* (**b**) according to Langmuir isotherms.

materials

MDPI

Table S1. The chemical composition (mg L^{-1}) of the aqueous suspensions of the studied carbons.

	Ag	Al	As	B	Ba	Be	Bi	Ca
C	<0.001	<0.005	0.0094	<0.01	0.0011	<0.0005	<0.0005	0.054
CN	<0.001	<0.005	0.0098	<0.01	0.0009	<0.0005	<0.0005	<0.05
CS	<0.001	<0.005	0.0097	<0.01	0.0013	<0.0005	<0.0005	<0.05
CNS	<0.001	<0.005	0.0096	<0.01	<0.0005	<0.0005	<0.0005	<0.05
CN-Fe	<0.001	<0.005	0.0097	<0.01	<0.0005	<0.0005	<0.0005	<0.05
C-Fe	<0.001	<0.005	0.0096	<0.01	0.0025	<0.0005	<0.0005	<0.05
CS-Fe	<0.001	<0.005	0.0094	<0.01	<0.0005	<0.0005	<0.0005	<0.05
CNS-Fe	<0.001	<0.005	0.0092	<0.01	<0.0005	<0.0005	<0.0005	<0.05
	**Cd**	**Co**	**Cr**	**Cu**	**Fe**	**Hg**	**K**	**Li**
C	<0.0003	<0.0002	0.068	0.0399	<0.02	<0.0001	0.75	<0.001
CN	<0.0003	<0.0002	0.070	0.0477	<0.02	<0.0001	0.71	<0.001
CS	<0.0003	<0.0002	0.072	0.052	<0.02	<0.0001	0.70	<0.001
CNS	<0.0003	<0.0002	0.068	0.0556	<0.02	<0.0001	0.67	<0.001
CN-Fe	<0.0003	0.0014	0.078	0.0587	<0.02	<0.0001	0.64	<0.001
C-Fe	<0.0003	0.00026	0.068	0.0595	<0.02	<0.0001	0.63	<0.001
CS-Fe	<0.0003	0.00093	0.069	0.0602	<0.02	<0.0001	0.63	<0.001
CNS-Fe	<0.0003	<0.0002	0.071	0.0607	<0.02	<0.0001	0.62	<0.001
	**Mg**	**Mn**	**Mo**	**Ni**	**PO4**	**Pb**	**Sb**	**Se**
C	0.015	<0.003	<0.0003	0.0016	<0.0061	<0.0001	<0.0002	<0.01
CN	<0.001	<0.003	<0.0003	0.0016	<0.0061	<0.0001	<0.0002	<0.01
CS	0.0011	<0.003	<0.0003	0.0016	<0.0061	<0.0001	<0.0002	<0.01
CNS	<0.001	<0.003	<0.0003	0.0016	<0.0061	<0.0001	<0.0002	<0.01
CN-Fe	<0.001	0.0032	<0.0003	0.0017	<0.0061	<0.0001	<0.0002	<0.01
C-Fe	0.0056	<0.003	0.00045	0.0017	<0.0061	<0.0001	<0.0002	<0.01
CS-Fe	<0.001	<0.003	<0.0003	0.0017	<0.0061	<0.0001	<0.0002	<0.01
CNS-Fe	<0.001	0.0060	<0.0003	0.0017	<0.0061	<0.0001	<0.0002	<0.01
	**SiO_2**	**Sr**	**Te**	**Ti**	**Tl**	**V**	**W**	**Zn**
C	0.032	0.0020	<0.01	<0.02	<0.0001	0.0184	<0.0003	<0.001
CN	0.024	0.0012	<0.01	<0.02	<0.0001	0.0186	<0.0003	<0.001
CS	0.023	0.00078	<0.01	<0.02	<0.0001	0.0189	<0.0003	<0.001
CNS	<0.02	0.0013	<0.01	<0.02	<0.0001	0.0185	<0.0003	<0.001
CN-Fe	<0.02	0.00030	<0.01	<0.02	<0.0001	0.0190	<0.0003	<0.001
C-Fe	<0.02	0.00064	<0.01	<0.02	<0.0001	0.0189	<0.0003	<0.001
CS-Fe	<0.02	0.00062	<0.01	<0.02	<0.0001	0.0189	<0.0003	<0.001
CNS-Fe	<0.02	0.00052	<0.01	<0.02	<0.0001	0.0188	<0.0003	<0.001
